# Brain-Inspired Spiking Neural Network Controller for a Neurorobotic Whisker System

**DOI:** 10.3389/fnbot.2022.817948

**Published:** 2022-06-13

**Authors:** Alberto Antonietti, Alice Geminiani, Edoardo Negri, Egidio D'Angelo, Claudia Casellato, Alessandra Pedrocchi

**Affiliations:** ^1^Neurocomputational Laboratory, Department of Brain and Behavioral Sciences, University of Pavia, Pavia, Italy; ^2^Nearlab, Department of Electronics, Information and Bioengineering, Politecnico di Milano, Milan, Italy; ^3^Brain Connectivity Center, IRCCS Mondino Foundation, Pavia, Italy

**Keywords:** point neuron model, neurorobotic architecture, active whisking, trigeminal ganglion, trigeminal nuclei, facial nuclei, central pattern generator (CPG), vibrissae

## Abstract

It is common for animals to use self-generated movements to actively sense the surrounding environment. For instance, rodents rhythmically move their whiskers to explore the space close to their body. The mouse whisker system has become a standard model for studying active sensing and sensorimotor integration through feedback loops. In this work, we developed a bioinspired spiking neural network model of the sensorimotor peripheral whisker system, modeling trigeminal ganglion, trigeminal nuclei, facial nuclei, and central pattern generator neuronal populations. This network was embedded in a virtual mouse robot, exploiting the Human Brain Project's Neurorobotics Platform, a simulation platform offering a virtual environment to develop and test robots driven by brain-inspired controllers. Eventually, the peripheral whisker system was adequately connected to an adaptive cerebellar network controller. The whole system was able to drive active whisking with learning capability, matching neural correlates of behavior experimentally recorded in mice.

## 1. Introduction

A fundamental question in system neuroscience is to identify how peripheral sensory stimuli are processed in multiple brain regions showing specific neuronal activity. Rodent whisker-mediated touch system is a structurally well-known system that gives rise to complex adaptive behaviors (Adibi, 2019). Specifically, the rodent whisker system represents an efficient combination of active perception and sensorimotor integration, in which self-generated movements are used to actively sense their environment, i.e., scanning the surroundings to collect behaviorally-relevant information. Rodents have specialized muscles in their mystacial pad to control the hair position (Moore et al., 2014). They rhythmically protract their whiskers, swiping the space surrounding the head and gathering information about the shape and position of objects around them.

In the rodent whisker system, the primary afferences come from the trigeminal ganglion (TG) and the efferences project to motoneurons in the facial nuclei (FN). There are no direct connections between the two; indeed, the innermost feedback loop is a di-synaptic reflex at the brainstem level, involving interneurons from the trigeminal nuclear complex (TN) (Nguyen and Kleinfeld, 2005; Bellavance et al., 2017). Outer loops in the whisker system involve multiple brain regions: the cerebellum, the midbrain (superior colliculus), and the forebrain (Bosman et al., 2011). The whisker-barrel loop is the most extensive and most studied cortical loop, involving the vibrissa primary sensory and motor cortex (McElvain et al., 2018). Therefore, this somatosensory system is ideal for investigating the link between circuitry and function and understanding the underlying neuronal mechanisms in sensory readout and information processing.

Computational models of this system can play a fundamental role in multi-scale investigations, from neuron to behavior, thanks to the availability of multi-scale experimental data in rodents for constraining and validating the models. In this work, we have developed a Spiking Neural Network (SNN) model able to process information encoded during whisking using a time coding representation of neuronal activity (Ghosh-Dastidar and Adeli, 2009; Ponulak and Kasiński, 2011; Brette, 2015; Tavanaei et al., 2019). While other models and kinds of artificial neural networks (e.g., rate-based or mean-field models) are very powerful tools, based on brain dynamics, we choose SNNs because they are closer to biological reality since they mimic the way information is coded and transmitted inside a real brain. Furthermore, spike timing is critical in brain dynamics and, therefore, in function generation. Thus, spike-based modeling strategies are needed to face this issue of whisking control and brain-inspired adaptation systems. In this work, each neuron in the network has been modeled with the most simplified spiking model, which is the Integrate & Fire model (I&F). SNNs can learn patterns of activity thanks to embedded plasticity models: here, we included a Spike-Timing Dependent Plasticity (STDP) model (Izhikevich and Desai, 2003; Izhikevich, 2007; Markram et al., 2011, 2012; Delattre et al., 2015) in the cerebellar circuit, which was inserted in the control system (outer loop) to test learning capabilities.

### 1.1. Neurorobotic Models of Rodent Whisking

Models of brain regions embedded in neurorobots allow us to reproduce the functional mechanisms of living beings in closed perception-action loops (Chen et al., 2017; Knoll, 2017). Various examples of neurorobots using biologically inspired whiskers have been implemented in the last years. Among them, it is worth citing the Whiskerbot, the SCRATCHbot, and the Shrewbot (Pipe and Pearson, 2015).

The Whiskerbot consists of a robotic platform constituted by a head sensory unit of 150 × 170 mm and a two-wheeled body. The head carries six whiskers per side arranged in rows of three. Analogue information from whisker deflection is converted in empirically-based spike trains. It can freely move in an environment, actively whisking and orienting toward salient stimuli using a neural network model of the superior colliculus (Pearson et al., 2007; Pipe and Pearson, 2015). The SCRATCHbot has a larger number of whiskers and degrees of freedom to position them in the environment. It was developed to reproduce different models of whisking pattern generation and actively explore its environment using a simple model of tactile attention (Pearson et al., 2010; Pipe and Pearson, 2015). Both these robots were further enriched by integrating the Shrewbot platform, which introduced algorithms able to detect texture and objects from an active whisker array (Pearson et al., 2011; Sullivan et al., 2012; Pipe and Pearson, 2015).

Real neurorobots are excellent test benches to challenge a neuro-inspired controller to demonstrate its capabilities, primarily because of the noise of the physical hardware and equipment, both intrinsic (non-ideal electronics sensors, limited spatio-temporal resolution, delays) and extrinsic (unexpected changes in the environment, external perturbing forces/torques, etc.). However, the implementation of physical neurorobots is complex and expensive, therefore limiting their adoption by neuroscientists to test computational models of brain circuitry. Besides, it is also challenging to replicate the obtained results without an exact replica of the equipment used. Finally, the brain-inspired circuit controlling the robot can have a limited complexity in terms of realism (neuronal models), the number of elements (neurons and synapses), activity (spike events), and functionality (e.g., short and long term plasticity rules) for the sake of limited computational load required for real-time computations.

In this paper, we have developed a biologically-inspired neurorobotic whisker system on a virtual mouse inside the Neurorobotics Platform (NRP) (Falotico and et al., 2017; Vannucci et al., 2017; Bornet et al., 2019; Corchado et al., 2019). This work focuses on reproducing the peripheral parts of the whisker sensorimotor system and integrating the sensory inputs with an adaptive cerebellar spiking controller to perform a spatial learning task.

## 2. Materials and Methods

In this section, the anatomy and physiology of the rodent whisker system are described, and for each peripheral component (active vibrissae, sensory pathway, motor pathway, and trigeminal loop) the neurorobotic implementation is reported. When building the whisking system, we tried to follow biological evidence, when available, or bioinspired principles while also achieving a compromise with computational constraints. For example, for neural population sizes, we followed information from literature about neuron type numbers and/or population size ratios while also considering that a more extensive network size would also increase the computational load of simulations. Then the protocol to test the whisking controller, including an adaptive cerebellar network, is described. It is tailored to the experimental paradigms used on mice to understand neural mechanisms of active whisking and reward-based learning. Finally, the software libraries and computing resources are reported.

### 2.1. Rodent Whisker System and Its Neurorobotic Implementation

Given the low number of degrees of freedom involved and the ease of making tests in laboratory conditions, the rodents whisker system has become a popular model for studying brain development, experience-dependent plasticity, active sensation, motor control, and sensorimotor integration (Bosman et al., 2011; Moore et al., 2014).

We have implemented the physical and the neural elements that constitute the whisker system of a rodent. The first step was the implementation of active whiskers (or vibrissae) in the mouse robot, making them controllable and allowing the reading of dynamic and kinematic parameters and information about the contact with external objects. Then, it was necessary to read inputs from the simulated environment and encode them realistically in the behavior of vibrissal afferents. Once unprocessed data were gathered from afferents, further elaboration steps were carried out. Finally, these processed signals were used to directly control the motor actions, thus closing the first sensorimotor feedback loop or extracting higher-level information such as the phase of the whisking when a contact happened.

#### Active Vibrissae

Vibrissae are long and sensitive hairs common to most mammals, including all primates except humans (Horn, 1970). Mystacial vibrissae grow on the mystacial pad, located at the sides of the animal snout, and have a significant role in tactile spatial sensing and object discrimination (Brecht et al., 1997).

##### Neurorobotic Implementation

**Figure 1:**
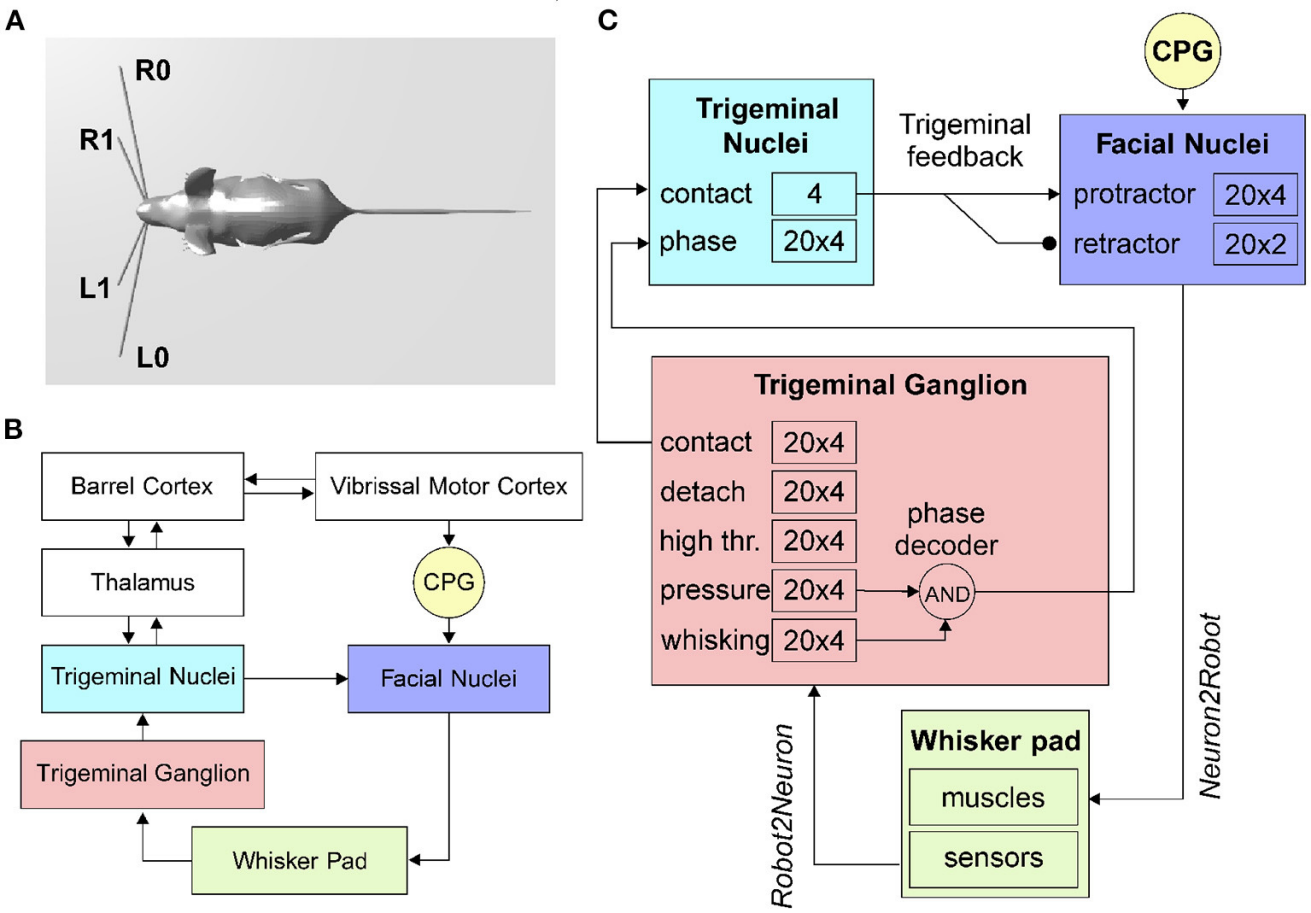
The rodent whisker system. **(A)** Virtual robotic mouse implemented in the NRP, with two whiskers per side. L0 and R0 are the lower left and right whiskers, L1 and R1 are the upper whiskers. **(B)** Block diagram of the rodent whisker system, including sensory and motor pathways, and its integration with higher-order areas (thalamus and cortex). **(C)** SNN implementation of the mouse peripheral whisker system; numbers in each block represent the size of the neural populations included in that brain region. Arrows represent excitatory connections, circles inhibitory connections.

To implement sensible whiskers in the mouse robot model inside the Neurorobotic Platform, we started from the HBP Mouse Robot v2 (dimensions: 140 cm from the nose tip to the end of the tail, 35 cm width, 35 cm height). The 3D models of the whiskers were defined as rigid cylinders: two right and two left whiskers, anchored to the mouse nose and with two different lengths and roll angles (Figure 1A). The lower whiskers (L0 and R0 for left and right whiskers, respectively) are longer (50 cm each) and are rotated of 11°, while the upper whiskers (L1 and R1) are shorter (25 cm each) and are rotated of 22°. All whiskers have a diameter of 1 cm, and their position can be independently controlled setting a torque at the revolving joints that link them to the mouse nose.

#### Sensory Pathway

In the rodent whisker system (Figure 1B), the primary afferents have their nucleus located in the trigeminal ganglion.

When an object enters the peri-personal space around a rodent's head, it will be sensed by the moving whiskers, and neuronal signals encode its position and shape. This can be done directly by the primary afferents or after some processing inside inner brain structures. The two information flows run in parallel and guarantees redundancy and a different level of elaboration. The way the position of an external object is represented inside the vibrissal system of rodents is called vibrissal location coding (Ahissar and Knutsen, 2016).

The spatial organization of whiskers in the mystacial pad varies between mammals but is quite similar between rats and mice (Brecht et al., 1997). Rats' and mice's whiskers are aligned in five rows, the upper two have four whiskers each, while the lower three counts seven whiskers each (Bosman et al., 2011). The whisker length increases exponentially from rostral to caudal in each vibrissal row (Brecht et al., 1997).

In the vibrissal follicle, three types of mechanoreceptors are present: Merkel cells, lanceolate endings, and free nerve endings (Bosman et al., 2011). Merkel cells are slowly adapting mechanoreceptors, and they mostly signal ongoing movements, while lanceolate endings are rapidly adapting and respond to fast changes.

Mechanoreceptors surrounding each whisker transmit sensory information through cells whose bodies are located in the TG. Each neuron sends signals from a single vibrissa, while 100–200 TG neurons innervate each follicle (Leiser and Moxon, 2007).

Szwed et al. (2003) induced artificial whisking in rats and measured the activity of TG cells. According to their results, TG neurons can be classified into distinct categories based on their responses to whisking in air and against an object:

- Touch cells responding only when the whisker touches an object, they can be further divided into contact cells, responding only at the beginning of the contact, detach cells, only at the end of the contact, and pressure cells with a tonic force-dependent response.
- Whisking cells responding only on whisker movement and not on object contact (if the contact does not affect the movement of the follicle).
- High threshold cells responding only to strong mechanical stimulations.

##### Neurorobotic Implementation

The Neurorobotics Platform allows connecting the environment to the robot sensors and actuators using so-called transfer functions. These Python functions define to and from which ROS topics and neural populations read and write. They can be defined as *Robot*2*Neuron* (sensory) or *Neuron*2*Robot* (motor) according to the direction of the information flow.

The transfer function written to implement the follicle sensors is of the *Robot*2*Neuron* kind since it reads the information on whisker mechanical status and position and then processes the data to obtain: possible contacts of a whisker against an object, the contact distance from the snout, the whisker angular position. The input transfer function is connected to the trigeminal ganglion neurons (Figure 1C), divided into five populations (TG pressure, TG high threshold, TG contact, TG detach, and TG whisking cells). We know that each follicle (i.e., each whisker) is innervated by 100–200 primary afferents (i.e., TG cells). Therefore, we have used 100 TG nuclear cells for each whisker, equally divided in contact, detach, high threshold, pressure, and whisking, each one implemented with 20 neurons (Szwed et al., 2003).

TG high threshold cells fire with a fixed rate when the contact is very close to the snout (<2 cm), constituting *de-facto* a labeled line encoding for proximity. TG whisking cells encode the current whisker position: each neuron has a Gaussian-shaped sensitivity and fires when the whisker position is within a narrow range around its maximum sensitive angle.

#### Motor Pathway

The head of a rodent exploring its peri-personal space is constantly moving, side-to-side and up-and-down, while its nose moves side-to-side, and the whiskers scan back-and-forth. These movements have a rhythmic component that is phase-locked to sniffing. The whisking frequency varies within ranges, with a mean value of 7 Hz (in rats) and 11 Hz (in mice) (McElvain et al., 2018).

The vibrissae representation in the primary motor cortex occupies around 20% of the motor cortical area. Although there is no accepted topographic map, some studies obtained single-whisker responses. In contrast, others observed how the number of whiskers showing evoked movements changes with the level of anaesthesia used. *In-vivo* single-cell microstimulation consistently evoked multi-whisker movements. There is strong evidence that the primary motor cortex indirectly controls the muscle activity projecting to brainstem premotor networks, acting as central pattern generators (CPG) (Schwarz and Chakrabarti, 2015).

Motor neurons controlling muscles of the whisker pad are located in the lateral FN and send motor commands *via* the facial nerve. About 80% of the FN neurons evoking whisker movements induce protractions of a single whisker and about 20% the retraction of multiple whiskers (Bosman et al., 2011).

##### Neurorobotic Implementation

The CPG has been implemented in the robot mouse as a single neuron. Controlled by a *Robot*2*Neuron* transfer function, the CPG neuron emits regular spikes at a constant frequency in the lower-theta band (4*Hz*). It is connected with excitatory synapses to both protractors and retractors neurons, with delays of 1 and 50*ms* respectively, in order to generate a rhythmic whisking movement.

Facial nuclei (Figure 1C) are divided into protractors and retractors. Protractors have been implemented with four populations of 20 neurons, where each population controls one whisker (L0, L1, R0, and R1). There are just two populations for retractors, one for each side (one population for L0 and L1, and one for R0 and R1). Sizes of populations are based on biological evidence. About 25–50 motoneurons innervate each intrinsic capsular muscle (Bosman et al., 2011). Given the ratios between protractors and retractors described above, we choose to have 30 facial nuclei per whisker (20 protractors and 10 retractors).

The spiking activity of protractors and retractors is then transformed into a torque signal, applied to each whisker, using (1).


(1) $$\begin{array}{r} torque\left(t\right)=\alpha_{pro}\cdot FR_{pro}\left(t\right)-\alpha_{ret}\cdot FR_{ret}\left(t\right) \end{array}$$

Where *FR*_*pro*_(*t*) and *FR*_*ret*_(*t*) are the instantaneous firing rates of protractors and retractors, respectively (in Hz), while α_*pro*_ and α_*ret*_ are constant gains, set to 1.5·10^−3^ Nm/Hz and 1.0·10^−3^ Nm/Hz, respectively. The instantaneous firing rates of a population are computed as the number of spikes in time bins of 10 ms normalized by the number of cells. With 20 neurons, the instantaneous firing rate can range from 0 to 100 Hz, in steps of 5 Hz.

#### Trigeminal Loop

The trigeminal loop in the brainstem is a second-order loop and is the most peripheral of the various loops constituting the vibrissal sensorimotor system. On the afferent side, neurons in the TG gather information from the follicles and project with excitatory synapses to the trigeminal nuclei complex. On the efferent side, subcortical whisking centers and CPG send motor commands to the motoneurons in the FN (Nguyen and Kleinfeld, 2005). Facial motoneurons driving muscles to protract the vibrissae receive a short latency input (7.5±0.4 ms) followed by synaptic excitation from neurons in TN. These connections result in a pull-push mechanism allowing for rapid modulation of vibrissa touch during exploration.

##### Neurorobotic Implementation

When a whisker touches an object, the physical simulator makes it bounce according to the physical properties of the simulated materials, producing a noisy contact signal. This offers us the possibility to apply the trigeminal feedback mechanisms previously described as a biologically inspired debouncing mechanisms. First, a neural population (TN contact) has been created in the trigeminal nuclei, four neurons (one per whisker) and taking from it excitatory, all-to-one connections (Figure 1C). Then, TN contact neurons were connected to the facial nuclei protractors with excitatory synapses having a 7.5*ms* delay (Bellavance et al., 2017) and inhibitory synapses to the retractors. This increases the joint torque sent to the colliding whiskers to impede their rebound and keep them in contact with the touched object. Unfortunately, there is no available information from biology about TN counts; therefore, we followed the principle of having fewer neurons than TG since TN is at a higher level in the sensory stream.

In the TN, we have included an additional population made of 20 neurons for each whisker that has to encode the phase of the whisking period at which the contact occurs. TG has been implemented as an array of coincidence detectors (phase decoder), one for each TG whisking neuron, gating them in a logical AND with TG pressure neurons. The result is a labeled-line encoding of the contact phase. The phase decoder is implemented by a transfer function that takes input from afferents in TG (pressure and whisking cells) and projects to the TN phase population in the trigeminal nuclei (Figure 1C). The same spike rate is propagated downward for each TG whisking cell only if pressure cells are firing. The phase information is needed for the precise localization of the object touched by the whiskers with respect to the mouse head.

Table 1 summarizes the connectivity between the different populations of the whisker system.

**Table 1:** Connectivity of the SNN whisker system model.

**Synaptic connection**	**Type**	**Number**	**Convergence**	**Divergence**
CPG-FN protractors	Excitatory	80	1	80
CPG-FN retractors	Excitatory	40	1	40
TG contact-TN contact	Excitatory	80	20	1
TN contact-FN protractors	Excitatory	80	1	20
TN contact-FN retractors	Inhibitory	80	1	20

### 2.2. Closed-Loop Learning Experiments

The peripheral components of the whisker system described above have been tested inside the Neurorobotics Platform in free whisking conditions. The mouse moved its whiskers in an empty environment or touching an object, and the spiking activity of FN, TG, and TN has been recorded to verify the proper functioning of the developed system.

#### Experimental Whisking-Based Object Localization Task

To provide a meaningful example of how the developed mouse whisker system can be used to build *in-silico* neurorobotic experiments, we reproduced an experimental study investigating the cerebellum's involvement in a whisking-based object localization task in head-fixed mice (Rahmati et al., 2014). Rahmati and colleagues tested two populations of mice, one wild-type (Control) and one knock-out (L7-PP2B), suffering from genetically impaired cerebellar plasticity. Water-deprived mice had to learn to locate a vertical bar in their whisker field and lick a water droplet (GO trial) within a time response window or to refrain from licking (NOGO trial) according to the bar position.

Both mouse populations started with high hit rates and high false alarm rates during the first sessions. After the first four training sessions, control mice showed faster learning capabilities, reducing their licking response to NOGO trials. Conversely, knock-out mice randomly reduced their licking, staying close to the guess rate (Rahmati et al., 2014). Therefore, they concluded that cerebellar plasticity has a crucial role in this sophisticated cognitive task requiring strict temporal processing.

#### Neurorobotic Implementation of the Whisking-Based Object Localization Task

In the NRP, a licking-like movement has been set for the virtual mouse: it has to raise its head and touch a shelf positioned just above. A vertical bar is placed in the left whisker field during GO trials, and if the mouse raises its head, a reward signal is triggered. During NOGO trials, the bar is on the right, and if the mouse raises its head it does not receive any reward.

The experiment is composed of short trials of 2 s, divided into GO and NOGO trials. The vertical bar is displayed in the mouse whisker field for 1 s, while the response window continues till the end of the trial.

Trials were grouped in sessions composed of 10 trials, 5 GO and 5 NOGO, performed in a randomized sequence. The neurorobotic experiment included 27 sessions, following the experimental protocol. In order to evaluate the learning of the controller, for each session, we recorded the percentage of correct responses in GO trials (“hit rate”) and the percentage of responses in NOGO trials (“false alarms”).

#### Cerebellar SNN Model

To investigate *in-silico* the role of cerebellar plasticity during the task, we integrated a well-established cerebellar-inspired SNN model into the whisker system described above. Recently, a detailed spiking neural network model of the cerebellar microcircuit proved able to reproduce multiple cerebellar-driven tasks (Casellato et al., 2014; Antonietti et al., 2016, 2019; Geminiani et al., 2017; Corchado et al., 2019). Here, we used the model to drive learning in the *in-silico* whisking-based object localization task.

**Figure 2:**
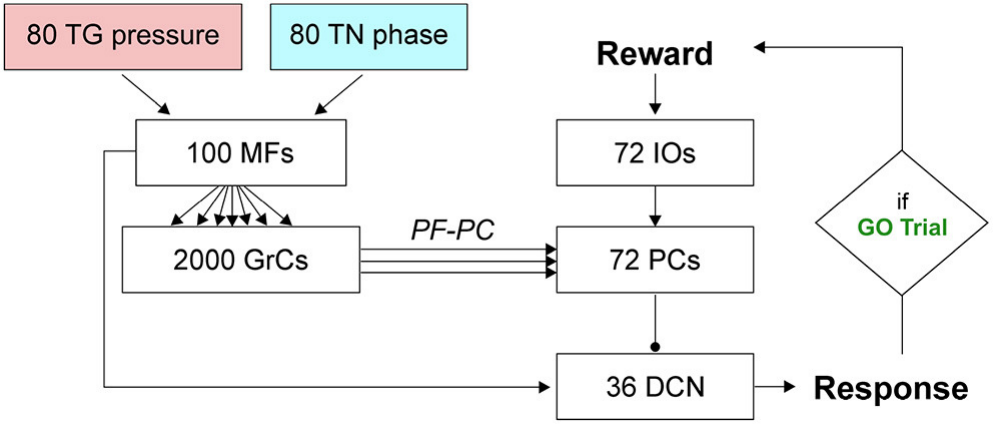
SNN implementation of the cerebellum. Whisking sensory signals are conveyed to the cerebellar MFs from TG pressure and TN phase neurons, while the reward signal during correct GO trials reaches the IO neurons; the cerebellum controls the output motor response (head movement) according to DCN activity (i.e., generation of a response, head raise, when the firing rate exceeds a set threshold). Arrows and circles represent excitatory and inhibitory connections, respectively.

The SNN cerebellar microcircuit (Figure 2) was populated with leaky Integrate&Fire neurons, distinguishing between different neural groups. Mossy Fibers (MFs), the input to the cerebellar module, encode the state of the body-environment system: the whisker current position and the localization of an eventual object, e.g., the cue signaling a GO trial. Therefore, MFs receive excitatory connections from TG pressure cells and TN phase cells. Granular Cells (GrCs) represent in a sparse way the input from the MFs. Inferior Olive neurons (IOs), the other input to the cerebellar module, encode the reward provided when a response is correctly generated (i.e., in a GO trial). In fact, this neural population responds to attention or surprise signals. Purkinje Cells (PCs) integrate the sparse information coming from the GrCs through the Parallel Fibers (PFs) with the one arriving from the climbing fibers, relaying IO spikes. Deep Cerebellar Nuclei (DCN), the only output of the cerebellar module, generate the response (i.e., the licking event). The firing rate of DCN is monitored, and a response is detected when the firing rate exceeds a pre-defined threshold (i.e., 80 Hz). The network structure and connectivity are reported in Figure 2 and Table 2.

**Table 2:** Connectivity of the SNN cerebellar model.

**Synaptic connection**	**Type**	**Number**	**Convergence**	**Divergence**
TG pressure-MFs	Excitatory	80	1	1
TN phase-MFs	Excitatory	80	1	1
MFs-GrCs	Excitatory	8,000	4	80
PFs-PCs	Excitatory	115,200	1,600	58
IOs-PCs	Teaching	72	1	1
MFs-DCN	Excitatory	3,600	100	36
PCs-DCN	Inhibitory	72	2	1

The cerebellar SNN model included one plasticity site, at the cortical level, between PFs and PCs, based on a well-known kind of STDP (Luque et al., 2011, 2016; D'Angelo et al., 2016). Synaptic weights between PF-PC plasticity are modulated by IO activity (IOs-PCs connections in Table 2 are indicated as “teaching”), depending on the difference between the pre- and post-synaptic firing times (Tolu et al., 2013; Geminiani et al., 2017; Ojeda et al., 2017). Long-Term Potentiation (LTP) and Long-Term Depression (LTD) are the two possible changes that each synaptic connection can undergo. Synaptic weights increase (LTP) whenever a PC only receives an input from a PF, while they decrease (LTD) when associated with IO inputs (Hansel et al., 2001; Jörntell and Hansel, 2006; Rasmussen et al., 2013; Ito et al., 2014; Hoxha et al., 2016). The learning rule can be formalized as in (2).


$$\begin{array}{r} \Delta W_{PF_{i}\to PC_{j}}\left(t\right) \end{array}$$

(2) $$=\{\begin{array}{ll} LTD\text{​​​​​​}\int_{-\infty }^{t_{IOspike_{j}}}\text{​}\text{​​​​​​​}K(t-x)\delta_{PF_{i}}(t-x)\text{d}x & {\text{if PC}}_{\text{j}}{\text{ is active and t=t}}_{{\text{IOspike}}_{\text{j}}}\\ LTP & {\text{if PC}}_{\text{j}}\text{ is active and t}\neq {\text{t}}_{{\text{IOspike}}_{\text{j}}} \end{array}$$

where:


(3) $$\delta_{PF_{i}}(s)=\{\begin{array}{ll} 1\; & {\text{if PF}}_{i}\text{ is active at time }s\\ 0\; & \text{otherwise } \end{array}$$

and the kernel function is:


(4) $$K(z)={\text{e}}^{-(z-t_{0})}{\left(sin(2\pi (z-t_{0}))\right)}^{20}$$

where *t*_*IOspik*_*e*__*j*__ is the time when *IO*_*j*_ emits a spike; *K*(*z*) is the kernel function, which has its peak at *t*_0_ (100 ms) before *t*_*IOspik*_*e*__*j*__. Its convolution with the PF spike train is integrated in the time window up to the *t*_*IOspik*_*e*__*j*__ to account for the time correlation between PF (state) and IO (error/reward) spikes in the LTD (3). The plastic learning rule is characterized by two constants, *LTP* and *LTD*, which regulate the amount of synaptic change. These constants cannot be directly computed from physiological data, but they have been set to values tuned in related modeling studies (*LTP* = 0.01, *LTD* = -0.03) (Antonietti et al., 2019). A PC is active at a certain instant when it produces a spike in that instant. Similarly for a PF, i.e., it is active when the granule cell to which it belongs fires at that instant. The learning rule is event-based and therefore is evaluated every time there is a spike on *PC*_*j*_. In fact, this is the mandatory condition of having LTD [upper branch of (3)] or LTP [lower branch of (2)].

#### Experimental and *In-silico* Cerebellar Impairment

**Figure 3:**
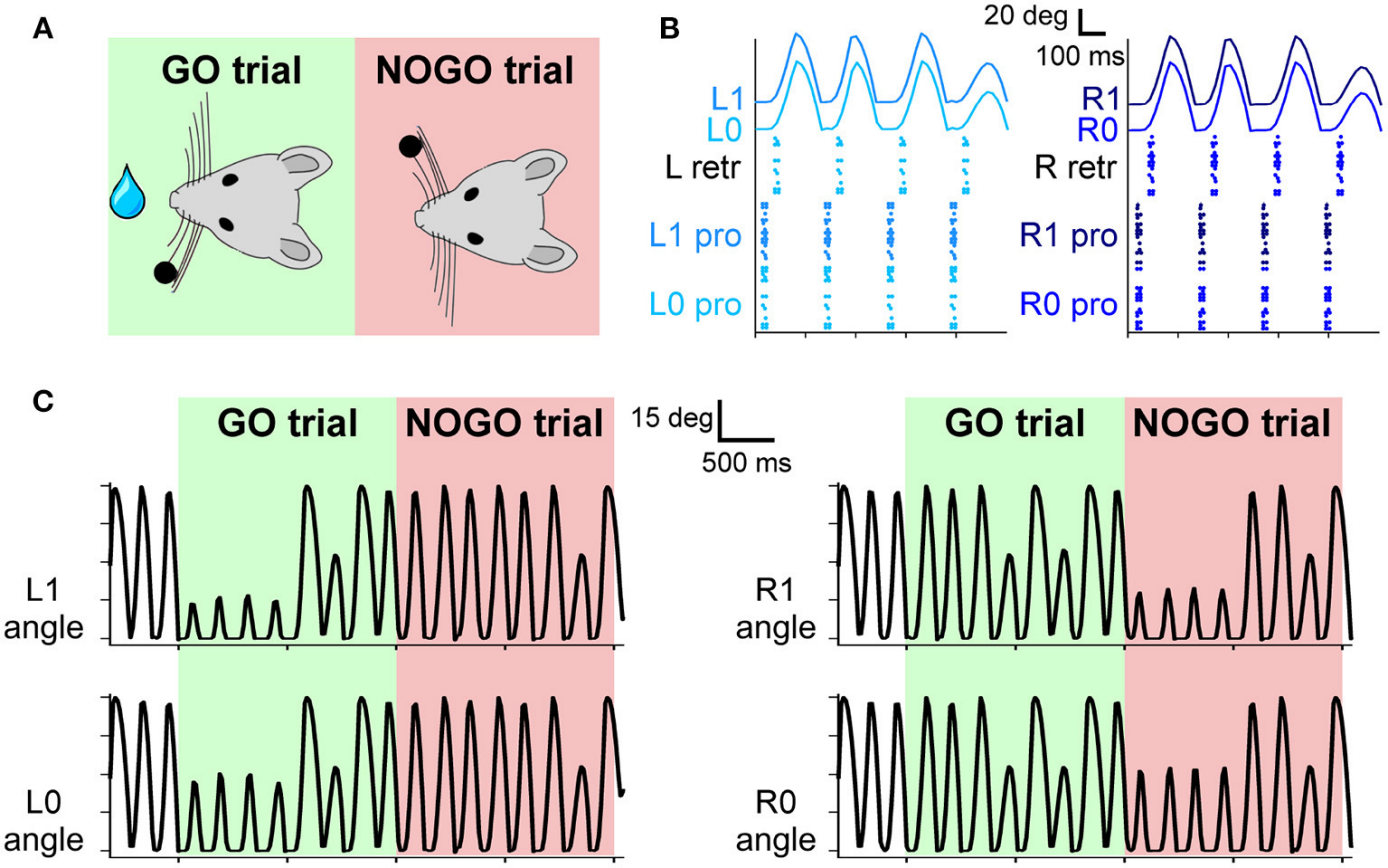
**(A)** The experimental protocol: during GO trials, a sensory cue (a small bar, depicted as a black dot) is placed in the left whisker field of the mouse. Correct responses lead to a reward (water drop). During NOGO trials, the sensory cue is placed in the right whisker field, and a response does not result in any reward. **(B)** Spiking activity of motor neurons for protraction and retraction during one trial. Twenty protractors neurons for each whisker and 20 retractor neurons for each side (L and R) fire under the control of the CPG neuron at 4 Hz. The resulting displacement of each whisker is depicted in the upper part of the panel. **(C)** Angular displacement of the four whiskers during one GO and one NOGO trial. During the GO trial, in the first second, left whiskers hit the sensory cue bar, placed in the left whisker field. On the other hand, during the NOGO trial, the right whiskers hit the sensory cue bar placed in the right whisker field.

PC-specific PP2B knock-out (L7-PP2B) mice show deficits in motor learning, consolidation, and procedural learning (Schonewille et al., 2010; Gao et al., 2012) while behaving normally in standard non-motor tasks (Galliano et al., 2013). In their experiment, Rahmati and colleagues tested how the impairment of the PF-PC LTP influenced the performance in the whisking-based object localization task (Figure 3A). They demonstrated that learning in L7-PP2B mice was severely impaired, indicating that this task can depend, at least to some extent, on cerebellar plasticity.

We recreated *in-silico* the impaired cerebellum dramatically reducing the constant *LTP* [see (3)] to 10% (*LTP*_*L*7−*PP*2*B*_ = 0.001). We repeated each experiment (i.e., 27 sessions, 10 trials each, therefore 270 trials) of the localization protocol 10 times, using the impaired cerebellar model. Then we compared the curves of hit rate and false alarms between L7-PP2B and control mice.

### 2.3. Hardware and Software

For the simulations, we have used a local installation of the NRP version 3.1, exploiting Python 3.8 (RRID:SCR_008394), Gazebo 11 (Aguero et al., 2015), and ROS Noetic (Quigley et al., 2009).

The simulation of the controller has been done with NEST, a software simulator for spiking neural networks (Gewaltig and Diesmann, 2007; Eppler et al., 2008; Plesser et al., 2015). We used NEST 2.18 (Jordan et al., 2019) (RRID:SCR_002963), interfaced through PyNN 0.9.5 (Davison, 2008) (RRID:SCR_002963).

All the simulations have been carried out on a Desktop PC provided with Intel Core i7-2600 CPU @ 3.40 GHz and 16 GB of RAM, running 64 bit Ubuntu 20.04.2 LTS.

## 3. Results

We successfully developed a SNN model of the sensorimotor peripheral whisker system, modeling trigeminal ganglion, trigeminal nuclei, facial nuclei, and central pattern generator neuronal populations. This peripheral SNN was embedded in a virtual mouse robot, and it was properly connected to an adaptive cerebellar SNN. The whole system was able to drive active whisking with learning capability, matching neural correlates of behavior experimentally recorded in mice.

### 3.1. Motor Pathway

The four whiskers are controlled by the motoneurons present in the FN. They are working under the control of a single CPG neuron, firing at 4 Hz, which rhythmically excites protractors and retractors neurons. Motoneurons spikes are then transformed into torques applied independently at each whisker. As shown in Figure 3B, during a free whisking period, the spiking pattern of the four groups of neurons is very similar, with a precise temporal alternation between protractors, causing the whisker to move forward, and retractors, pulling the whiskers back to the initial position. Whiskers' movements are slightly shifted with respect to the spikes due to the delays introduced by the conversion between spikes and torques and by the mechanical inertia of the whiskers. The mean firing rate of protractors and retractors neurons is 4 Hz, with a peak firing rate of 49 and 55 Hz, respectively. Figure 3C shows how the whisker trajectory changes during GO and NOGO trials. Namely, in GO trials, a bar is placed for 1 s in the left whisker field; therefore, whiskers L0 and L1 hit it, and their range of motion is reduced to ~15 degrees. The same behavior can be observed during NOGO trials for whiskers R0 and R1.

### 3.2. Sensory Pathway

**Figure 4:**
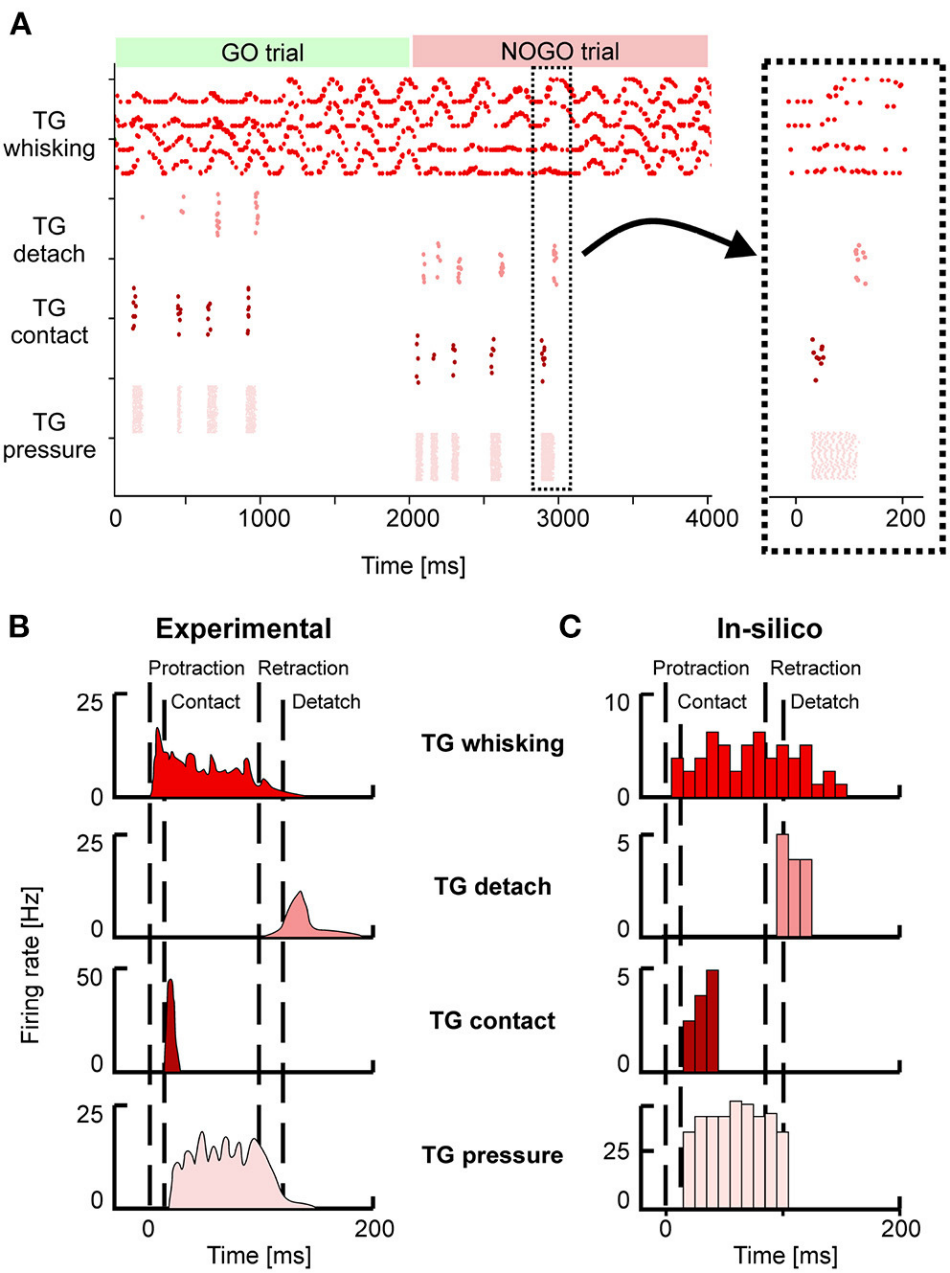
**(A)** Spiking activity of the Trigeminal Ganglion (TG) neurons during GO and NOGO trials. Each row represents the activity of one neuron, and different shades of red are used to plot the activity of the four groups of TG neurons. The inset shows a magnified portion of the full scatterplot, focusing on a single protraction-retraction movement of the whiskers. **(B)** Firing rates of TG populations were measured experimentally during a single protraction-retraction movement, as reported in Ahissar and Knutsen (2016). Vertical dashed lines represent the four main events: the start of the protraction, contact of the whisker against an object, the start of the retraction, detach of the whisker from the object. **(C)** Firing rates were recorded from the simulation of the SNN model of TG populations. The length of each bin is 10 ms. Colors are the same as **(A,B)**.

Neurons in the TG and TN compose the sensory pathway, and TG neurons do the first elaboration stage. Each group of TG shows specific activity patterns depending on its function (Figure 4A). TG whisking neurons follow the angular profile of each of the four whiskers; it is possible to notice the differences between GO and NOGO trials, where left and right whiskers change their spatial profile when hitting the bar in the first second of each trial. Their mean firing rate is 4 (± 2) Hz. TG contact neurons fire at ~5 Hz when the whisker hits the bar, while TG detach neurons when the whisker is no longer in contact with the bar because the bar has been removed or because the whisker has been retracted. TG pressure neurons are active for the whole duration of the contact between the whisker and the bar (27 ± 18 Hz). In the SNN model, we have included an additional population, TG high threshold neurons, which are activated when the contact happens close to the nose of the robot (<2 cm). However, in our protocol, the bar is placed at a higher distance, therefore, those neurons were never activated.

Figures 4B,C provide a direct comparison between the firing rates of the different TG populations during one whisker movement. Figure 4B has been adapted from Ahissar and Knutsen (2016), while in Figure 3C the firing rates of the neurons in a specific trial (the magnified inset from Figure 4A) have been computed with bins of 10 ms. It is possible to appreciate that *in-silico* TG neurons show a behavior comparable to the one of biological neurons, especially for the timing of their response with respect to the events of protraction, contact, detach, and retraction.

### 3.3. Learning Performance

We have shown that the SNN representing the sensorimotor whisker system can encode the sensory and motor signals exchanged with a robotic mouse in a biologically realistic way. To demonstrate how this system can be used to recreate a complex behavioral test, we connected the whisking sensory system to an adaptive SNN, and we challenged the integrated system in the object localization experiment proposed by Rahmati et al. (2014).

**Figure 5:**
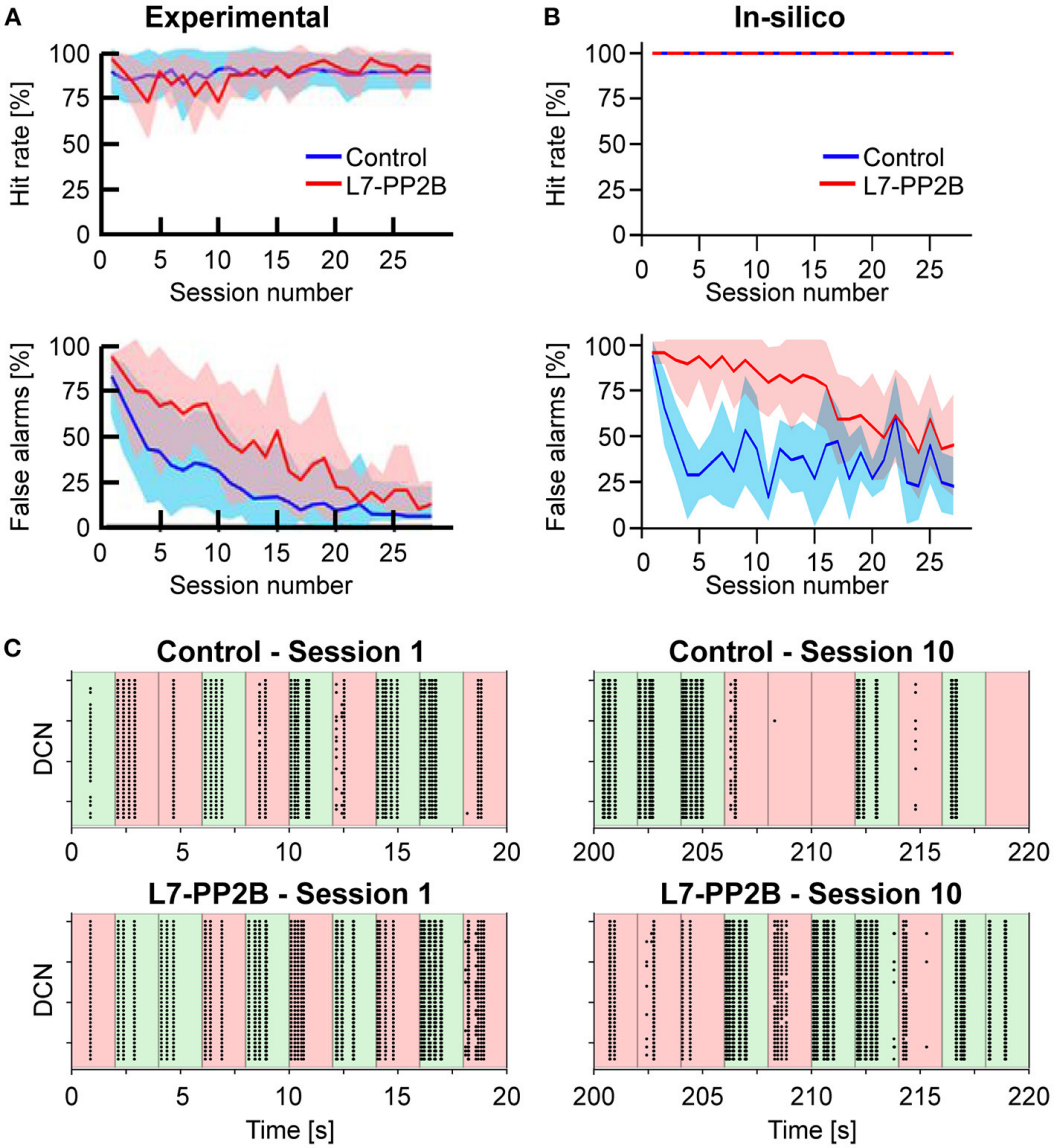
**(A)** Learning curves recorded in the experiment performed by Rahmati et al. (2014). The upper row shows the Hit rate (i.e., the percentage of correct responses in GO trials) along with sessions, where each session is composed of 10 trials. The lower row shows the False alarms (i.e., the percentage of incorrect responses in NOGO trials) along with sessions. The blue and red curves show the mean values for control animals and knock-out (L7-PP2B) mice. Shaded areas show the standard deviation. **(B)** Learning curves recorded from the *in-silico* experiments (10 control and 10 knock-out models). Colors are the same as in **(A)**. **(C)** Spiking activity of the DCN neurons during GO (green) and NOGO (red) trials, in the first session (left column) and after 10 sessions of training (right column). The first row reports the activity of one Control simulation, while the second row reports one knock-out simulation. Each dot is a spike of one of the 36 DCN in the cerebellar network. The order of GO and NOGO trials is randomized for each session and simulation, but all sessions have 5 GO and 5 NOGO trials.

The aim of the mouse is to lick during the GO trials and to refrain from licking during the NOGO trials, distinguishing between the two conditions according to the position of a bar placed into their whisker field. Figure 5B reports the percentages of correct licks in GO trials and the number of incorrect licks in NOGO trials. The reference behavioral data recorded in animals are reported in Figure 5A.

Considering control animals, it is possible to see that mice lick continuously in the first sessions (i.e., hit rate and false alarms are both close to 100%) without distinguishing between GO and NOGO trials. While the experiment proceeds, the animals learn to refrain from licking during the NOGO trials. In fact, the percentage of false alarms decreases toward 0%. At the same time, the rate of correct licks remains close to 100%.

Control and L7-PP2B mice differed in their learning skills. Both started the training with high hit and false alarm rates. As a result, they performed close to the guess rate. However, during the subsequent sessions, control mice consistently increased accuracy, specifically reducing their response to NOGO trials. In contrast, L7-PP2B mice continue for more sessions to not discriminate between GO and NOGO trials. In the later sessions, also L7-PP2B diminished their licks in the NOGO trials. Still, their learning trajectories remained noisier than those of controls, taking a longer time to reach high-performance levels. This observation suggested that a functional LTP mechanism was essential to obtain a superior ability to rapidly discriminate between GO and NOGO cues and then respond accordingly.

The performances and the learning trajectories of control and L7-PP2B *in-silico* models (Figure 5B) are similar to their biological counterparts. While both models perfectly recognize GO trials, the control model learned to refrain from licking in NOGO trials faster and stably. The variability present between the 10 different tests is due to the various sequences of GO and NOGO trials, which were randomly extracted for each session.

Looking at the spiking activity of the cerebellar network, particularly in the DCN population (Figure 5C), which drives the response of the mouse, it is possible to appreciate the different evolution in the spiking patterns. Control and L7-PP2B simulations have a similar activity in the DCN during the 10 trials of the first session, the neurons fire regardless of the input arriving from the whisker system (right or left contacts), and therefore DCN generate a response during both GO and NOGO trials. After 10 sessions of training, the Control simulation shows intense DCN activity during GO trials and weak or null activity during NOGO trials, proving that the cerebellar network has learned the association between left/right stimulation with the presence/absence of the reward. This behavior is impaired for the L7-PP2B simulation, in fact, there are still several DCN that are firing during both GO and NOGO trials, thus causing a high False alarm rate.

Each session took about 140 min for a simulated time of 540 s (i.e., 270 trials of 2 s each), with a slow down with respect to the real-time equal to ~15 times. The maximum RAM consumption was equal to ~15 GB.

## 4. Discussion and Conclusions

We developed a spiking neural model of the mouse whisker system, covering both sensory and motor pathways and their interconnections. The implemented system considers the different roles that groups of cells have at the various stages of sensorimotor processing, providing coding for complex information such as the object localization performed during the active whisking. This system, properly connected to an adaptive cerebellar-inspired spiking network, reproduced complex *in-vivo* experiments using the neurorobotics platform.

The paper focuses on the methodological aspects of the neurorobotics implementation of the whisker system, including its bioinspired components and their interconnections. In addition, we aimed at developing and making available a spiking neural network controller that can be incorporated into a virtual robotic platform. Specifically, the developed whisker system model can be exploited to study the signal transmission in rodent whisking, which is a relevant paradigm of sensorimotor integration. We have then included an exemplary application to show how the system can be used to emulate an experimental protocol that involves the whisker system.

The peripheral whisker system showed appropriate discharge patterns as in *in-vivo* experimental recordings during whisking, in precise time windows of exploration and object interaction and depending on which side the stimulus was presented within the whisker field.

The peripheral system, when wired to a cerebellar SNN with plasticity and tested in an object-localization task, was able to reduce the number of useless responses along a sequence of trials (triggered by the NOGO trials) which did not correspond to any reward. However, this learning curve was slowed down when the plasticity parameter (LTP rate) of the cerebellar SNN was strongly reduced, as in knock-out mice recorded experimentally.

The integrated circuit, entirely made of spiking neurons, proved the good integration of different ways of neural coding. In fact, while the main parameter correlating response patterns to behavior was the average firing frequency of the DCN population, other elements of the whisker system used a variety of encoding strategies. For instance, the time-coded activity of TG contact and TG detach cells. Also, the TG whisking cells encode the current whisker position by means of their Gaussian-shaped sensitivity.

The model here proposed can be used as a reference for future advanced neurorobots and neuroscience *in-silico* experiments to investigate the role of cerebro-cerebellar loops and cerebellar physiology in whisking protocols. We invite computational neuroscientists to leverage our system to implement *in-silico* experiments to shed light on unsolved scientific questions. There are many possible experiments and manipulations that can be done on the proposed system (e.g., lesion studies with the deactivation of one or more neural populations). However, it is advisable to carefully think of what are experiments having a feasible counterpart in the biological world.

### 4.1. Limitations and Future Challenges

Considering the mechanical aspects, a limitation of the physical simulator (Gazebo) regards the properties of the materials used. For example, rodents rely on whiskers bending to recognize the shape of objects and on their resonance frequencies to detect textures (Neimark et al., 2003; Jones et al., 2009), but the current state of the simulators used by the NRP supports only rigid bodies. Using only stiff whiskers makes object recognition tasks more difficult unless maybe using large arrays of finely spaced whiskers. Therefore, this work focused on extracting only spatial information, which can be easily performed with just rigid whiskers, and not more sophisticated features of the touched object.

Much of the work on the whisker system consisted in the encoding of information in TG primary afferents, ignoring all of the internal brain structures involved in the whisker system, in particular, the somatotopic mapping emerging in the TN and propagated in the thalamocortical system. Loops between the thalamus and cortex have been cited as a possible location for mechanisms decoding phase information with the use of neuronal phase-locking loops. A possible future development can be exploring other loops in the brainstem outside the TN, such as the ones involving the superior colliculus and their interactions with attention and foveation (Kaneshige et al., 2018).

The absence of bidirectional interactions with the sensorimotor cortex does not allow to study voluntary modulation of the whisking action. However, lesion studies have shown that primary sensory cortex ablation prior to learning did not affect whisking task acquisition (Hong et al., 2018). The interaction with higher-level brain areas does not seem to be needed with a simple free whisking task like the one we presented. Given that the model is based on open-source simulators and the code is made available to the community, neuroscientists could easily integrate spiking models of the sensorimotor cortex in our model in future work to investigate voluntary control of whisking movements. In addition, it is known that multiple brain areas interact to generate behaviors. However, brain models including only some of the involved circuits can help clarify the specific roles of these subcircuits, isolating their contribution to the output behaviors.

The work on the cerebellar control mechanisms was mainly limited by the long simulation times of the NRP, which influenced the choice of network and learning parameters. Given the limited number of mossy fibers and granule cells (100 and 2,000, respectively), the cerebellar network showed a reduced generalization capability. The discrimination task, in this case, was between two very different conditions (object hit with the left or with the right whiskers). Mice have a higher resolution since they can recognize slightly moved objects or objects with different textures. With larger populations, training could make different sub-populations respond to other inputs, encoding for more complex features of the sensed environment. Future work can explore this hypothesis, making rigorous analyses on cell responses and optimizing the network size to the variety of input patterns.

We have chosen I&F neuron models as building blocks of the SNN network. However, nowadays, there are much more complex models, taking into account many mechanisms related to membrane potential and ionic currents (biophysical models; Hines and Carnevale, 2001) or more advanced I&F neuron models (Izhikevich, 2003; Geminiani et al., 2019). Even though these models are more accurate representations of the biological elements, their complexity would require too much computational power to simulate a network made of thousands of neurons, which would eventually prevent embedding these models as controllers in neurorobots. Therefore, the model we are proposing is a trade-off between computational efficiency and biological realism. Similarly, the system can work with more or fewer neurons, but the performances will change from both behavioral and computational points of view. More neurons will yield a higher resolution and a more precise frequency modulation, at the same time with an increase in computational complexity. The whisker system that we propose has numbers in the same order of magnitude as the biological entities, with some simplifications (e.g., only four whiskers) needed for feasible simulations.

NEST-based simulations offer a great possibility to develop and test biologically inspired models but require high-performance computing for large-scale models (Jordan et al., 2018) and therefore do not allow performances sufficient to control robots in real-world situations. This could enable testing the robustness of the bioinspired controller against common environmental noise, increasing the similarity with experimental results [e.g., the imperfect (<100%) hit rates accuracies achieved by experimental animals]. An already available solution to gain real-time performances can be to rely on spiking neural networks running on neuromorphic hardware. Very recently, a cerebellar-inspired model made of 97,000 neurons and 4.2 million synapses has been implemented on the neuromorphic platform SpiNNaker (Bogdan et al., 2021). This solution could be applicable if the plasticity rule used at PF-PC synapses, supervised by IO activity, will be implemented on this or other neuromorphic systems. On the other hand, the SNN whisker system here presented can be simulated on SpiNNaker chips since it has been developed using PyNN, which supports both NEST and SpiNNaker as simulators.

### 4.2. Conclusions

Neuroscientists have not fully uncovered the neural mechanisms for mouse whisking, but it is clear that it involves a complex architecture composed of multiple sensorimotor loops. In this work, we developed and tested a spiking computational model of the peripheral whisker system, reproducing the neural dynamics observed in its different components and embedded in a virtual mouse neurorobot controlled by a cerebellar SNN.

The virtual mouse enriched with this peripheral whisker system may be connected to more realistic multi-area brain models to show how these regions together may control the precise timing of whisker movements and coordinate whisker perception.

In the future, refined versions of the model could exhibit more advanced features, such as the recognition of surface textures, identification of movements of the touched object, or other complex touch-guided behaviors. In addition, from a technological perspective, neuromorphic implementations can speed up the computation until reaching real-time performances, allowing the possibility of embedding the whisker system in physical robots.

## Data Availability Statement

The datasets presented in this study can be found in online repositories. The computational models used for the simulation are publicly available at: https://github.com/alberto-antonietti/paper_whisking. Data generated from the simulations and Python codes to generate all the figures presented in this work are also available in the GitHub repository.

## Author Contributions

AA, AG, and CC designed the model and the computational framework. AA, AG, and EN carried out the implementation. AA and EN wrote the manuscript with input from all authors. AA, ED'A, CC, and AP conceived the study and were in charge of overall direction and planning. All authors discussed the results and commented on the manuscript.

## Funding

This research received funding from the European Union's Horizon 2020 Framework Program for Research and Innovation under the Specific Grant Agreement No. 785907 (Human Brain Project SGA2), Specific Grant Agreement No. 945539 (Human Brain Project SGA3), and the Voucher (CEoI 4 - Rodent microcircuits: RisingNet Whole-bRaIn rodent SpikING neural NETworks).

## Conflict of Interest

The authors declare that the research was conducted in the absence of any commercial or financial relationships that could be construed as a potential conflict of interest.

## Publisher's Note

All claims expressed in this article are solely those of the authors and do not necessarily represent those of their affiliated organizations, or those of the publisher, the editors and the reviewers. Any product that may be evaluated in this article, or claim that may be made by its manufacturer, is not guaranteed or endorsed by the publisher.

## Supplementary Material

Supplementary Video 1 The video makes it possible to see the mouse whisker system in action. First, the free whisking activity is shown: protraction and retraction of the four whiskers are driven by the neural activity of the Facial Nuclei. Then, the object-localization protocol is shown. The mouse touches with the whiskers the bar that is placed in its left (GO trials) or right (NOGO trials) whisker fields. A small shelf is positioned vertically above the mouse's nose, it changes color according to the trial condition (GO: green, NOGO: red). The DCN activity of the cerebellar circuit triggers the response of the mouse, which raises its head to touch the shelf. A reward is sent to the IO neurons of the cerebellar circuit only during GO trials (correct responses) while it is not sent during NOGO trials (false hits). In early trials, the mouse is responding during both GO and NOGO trials since it has not learned yet that only GO trials provide a reward. During late trials, the mouse learned to respond during GO trial only, refraining from raising its head during NOGO trials. Note that there are some glitches (e.g., the tremor of the 3D mesh representing the mouse body and the apparent random detachment of the whiskers) that are just rendering errors of the NRP while the actual physical behavior is correctly simulated.
